# Bone Marrow Ts65Dn Trisomy-Induced Changes in Platelet Functionality and Lymphocytopenia Do Not Impact Atherosclerosis Susceptibility in Mice

**DOI:** 10.3390/jcdd8090110

**Published:** 2021-09-13

**Authors:** Suzanne J. A. Korporaal, Ronald J. van der Sluis, Miranda Van Eck, Menno Hoekstra

**Affiliations:** 1 Division of BioTherapeutics, Leiden Academic Centre for Drug Research, Leiden University, Gorlaeus Laboratories, Einsteinweg 55, 2333 CC Leiden, The Netherlands; J.A.Korporaal-3@umcutrecht.nl (S.J.A.K.); R.J.van_der_Sluis@lumc.nl (R.J.v.d.S.); m.eck@lacdr.leidenuniv.nl (M.V.E.); 2 Department of Clinical Chemistry and Haematology, University Medical Center Utrecht, Utrecht University, Heidelberglaan 100, 3584 CX Utrecht, The Netherlands

**Keywords:** bone marrow, down syndrome, mouse model, lymphocytes, monocytes, macrophages, blood platelets, atherosclerosis

## Abstract

The genetic disorder Down syndrome is associated with a decreased susceptibility for atherosclerotic cardiovascular disease. Hematological and immune abnormalities occur frequently in Down syndrome patients. We evaluated, in a preclinical setting, the impact of a Down syndrome-like hematological/immune phenotype on atherosclerosis susceptibility. Hereto, hypercholesterolemic low-density lipoprotein receptor knockout mice were transplanted with bone marrow from either a trisomic Ts65Dn mouse or euploid wild-type control and subsequently fed a Western-type diet to induce the development of atherosclerotic lesions. T and B cell concentrations were markedly reduced in blood of Ts65Dn bone marrow recipients (*p* < 0.001). Expression levels of the pro-atherogenic scavenger receptor CD36 were respectively 37% and 59% lower (*p* < 0.001) in trisomic monocytes and macrophages. However, these combined effects did not translate into an altered atherosclerosis susceptibility. Notably, blood platelet numbers were elevated in Ts65Dn bone marrow recipients (+57%; *p* < 0.001), which was paralleled by higher platelet GPVI protein expression (+35%; *p* < 0.001) and an enhanced collagen-induced platelet activation (*p* < 0.001). In conclusion, we have shown that providing mice with a Down syndrome-like hematological profile does not change the susceptibility to atherosclerosis. Furthermore, our studies have uncovered a novel effect of the trisomy on platelet functionality that may be relevant in human clinical settings.

## 1. Introduction

The high frequency genetic disorder Down syndrome (trisomy 21; global incidence: 1 in 800 births) is associated with a relatively low susceptibility for the development of atherosclerotic cardiovascular disease, characterized by a narrowing of the vessel lumen due to subendothelial accumulation of cholesterol within macrophages. Postmortem analysis by Murdoch et al., in the 1970s, indicated that the coronary arteries, aorta and other large arteries of Down syndrome carriers were virtually free of atherosclerosis [1]. The atherosclerosis resistance phenotype was validated by Ylä-Herttuala et al. who detected significantly reduced cholesterol and calcium levels within coronary arteries of institutionalized Down syndrome carriers as compared to both institutionalized subjects suffering from non-Down syndrome-related mental handicaps and non-institutionalized control subjects without mental defects [2]. A more recent study on Down syndrome carriers with mild-to-moderate mental retardation has further proven that the atherosclerotic disease burden, i.e., as measured by the carotid intima-media thickness, is significantly lower in this specific population as compared to age-matched subjects without Down syndrome or mental retardation [3]. The relative atherosclerosis resistance of Down syndrome carriers does not seem to be related to an effect of the genetic disorder in the hyperlipidemia extent [3,4].

Down syndrome is the most common recognizable genetic syndrome associated with hematological and immune abnormalities. These include a relatively high predisposition for the development of acute myeloid and lymphoid leukemia, mild to moderate reductions in T and B cells, reduced T cell proliferation, and decreased neutrophil chemotaxis (reviewed by Ram et al. [5] and Satgé and Seidel [6]). Importantly, from the CANTOS trial it has become evident that alterations in the activity of the immune system can significantly impact cardiovascular disease burden in humans [7]. As such, a change in the immune status may possibly contribute to the relative atherosclerosis resistance observed in Down syndrome carriers. In the current study we therefore evaluated—in a preclinical setting—the impact of a Down syndrome-like hematological/immune phenotype on atherosclerosis susceptibility.

## 2. Materials and Methods

### 2.1. Mice and Bone Marrow Transplantation

Ts65Dn mice are trisomic for the segment of mouse chromosome 16 that is conserved in human chromosome 21 and display some classical features of human Down syndrome, i.e., growth retardation and cognitive deficits [8,9,10]. Bone marrow transplantation was performed at the Gorlaeus Laboratories of the Leiden Academic Centre for Drug Research essentially as described [11]. Female LDL receptor knockout mice with an average plasma total cholesterol level of ~200 mg/dL were irradiated and subsequently intravenously injected with 5 × 10^6^ bone marrow (BM) cells from a genotype-verified female trisomic or euploid wild-type control mouse of 12 and 9 months age that were obtained from the same crossing of a male C57BL/6 wild-type mouse and a Ts65Dn female mouse (Jackson Laboratories, Bar Harbor, ME, USA). Bone marrow recipient mice (*n* = 12 per group) were maintained on regular chow diet (RM3, Special Diet Services, Whitham, UK) for 8 weeks to recover from the transplantation and subsequently fed a Western-type diet, containing 15% (*w*/*w*) total fat and 0.25% (*w*/*w*) cholesterol (Diet W, Special Diet Services, Whitham, UK) for 6 weeks to exacerbate the hypercholesterolemia and induce the development of atherosclerotic lesions.

### 2.2. Generation of Bone Marrow-Derived Macrophages

Bone marrow precursors were differentiated into macrophages in DMEM supplemented with 10% fetal bovine serum, 2 mM glutamine, 20% L929 cell-conditioned media (as a source of M-CSF), and penicillin-streptomycin for 7 days and subsequently lysed using guanidinium thiocyanate for the following gene expression analysis.

### 2.3. Analysis of Gene Expression by Real-Time Quantitative PCR

Total RNA was isolated using a standard chloroform-phenol extraction method and reverse transcribed. Gene expression analysis was performed on an AB7500 Fast apparatus using real-time SYBR Green technology, essentially as described [12].

### 2.4. Blood Cell Analysis

Platelet and total white blood cell counts were routinely measured using an automated SYSMEX XT-2000iV Veterinary Heamatology analyzer (Sysmex Europe GMBH, Norderstedt, Germany) in blood collected from the tail. The presence of specific cell surface markers on leukocytes was detected by flow cytometry using the antibodies CD19-PerCP/Cy5.5, C36-PE, F4/80-FITC, CD11b-APC, CD8-PerCP, and CD4-PE from eBioscience on a FACSCalibur (Becton Dickinson, Mountain View, CA). Data were analyzed using Cell Quest software.

### 2.5. Histological Analysis of Atherosclerosis

Ten μm cryosections of the aortic root, serially collected starting from the tricuspid valves, were stained with Oil red O to identify atherosclerotic lesions. Mean lesion area from 5 sections per mouse was quantified by using a Leica image analysis system, consisting of a Leica DMRE microscope coupled to a camera and Leica QWin Imaging software (Leica Ltd., Cambridge, UK). Lesional collagen content was determined from Masson’s Trichrome Accustain (Sigma-Aldrich)-stained sections. Lesion analyses were performed in a blinded manner.

### 2.6. Platelet-Related Studies

To investigate platelet activation, we measured the ability of platelets to convert integrin αIIbβ3 to its high-affinity open conformation upon stimulation by different agonists. Hereto, equal volumes of blood (~900 µL) per mouse were collected by cardiac puncture in needles prefilled with 100 µL citrate. Twenty-five microliters of diluted and re-calcified whole blood was stimulated by ADP (Sigma-Aldrich, Zwijndrecht, the Netherlands), crosslinked collagen-related peptide (CRP-XL; Collagen Toolkits, Cambridge, UK), or protease activated receptor 4 (PAR-4)-activating peptide (Bachem, Weil am Rhein, Germany) for 15 min at 20 °C in the presence of PE-conjugated anti-active integrin αIIbβ3 (5 µL, clone JON/A; Emfret Analytics, Eibelstadt, Germany). Samples were fixed and active integrin αIIbβ3 was determined by flow cytometry. Platelet expression levels of GPVI (clone JAQ1; Emfret Analytics, Eibelstadt, Germany) were also assessed by flow cytometry in whole blood samples.

### 2.7. Data Analysis

Statistical analysis was performed using Graphpad Instat software (San Diego, CA, USA, http://www.graphpad.com (accessed on 10 September 2021)). Normality testing of the experimental groups was performed using the method of Kolmogorov and Smirnov (Graphpad Instat). The significance of differences was calculated using a two-tailed Student’s *t*-test or two-way analysis of variance (ANOVA) with the Bonferroni post-test, where appropriate. Probability values of *p* < 0.05 were considered significant.

## 3. Results and Discussion

**Figure 1:**
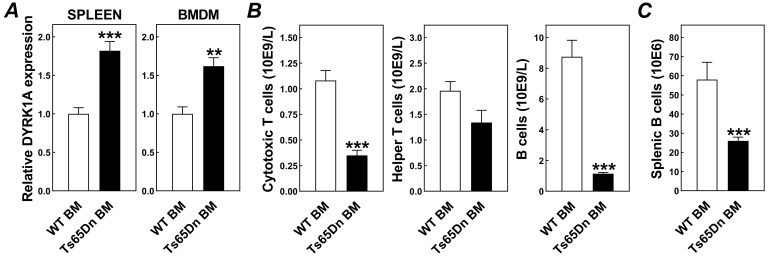
Successful induction of bone marrow trisomy is associated with lymphocytopenia. (**A**) Relative mRNA expression levels of the trisomic gene DYRK1A in spleens and bone marrow-derived macrophages (BMDM) and amounts of different lymphocyte subsets in blood specimens (**B**) and spleens (**C**) from Western-type diet-fed LDL receptor knockout mice that have received wild-type bone marrow (WT BM) or Ts65Dn bone marrow (Ts65Dn BM). Data represent means + SEM of respectively 10/12 mice (left figure in panel **A**) or 6 mice (all other figures) per group. ** *p* < 0.01, *** *p* < 0.001 versus WT BM.

Bone marrow transplantation is an established means of studying the specific impact of changes in the hematological/immune compartment on atherosclerosis outcome [13]. To pursue our research question, we therefore transplanted bone marrow from trisomic Ts65Dn mice and euploid wild-type control mice into atherosclerosis-susceptible hypercholesterolemic LDL receptor knockout mice that were subsequently fed a Western-type diet for 6 weeks to exacerbate the hypercholesterolemia and initiate the development of atherosclerotic lesions. Relative mRNA expression levels of the trisomy marker DYRK1A were 1.6- to 1.8-fold higher (*p* < 0.001) in bone marrow-derived macrophages and spleens from Ts65Dn bone marrow recipients as compared to wild-type bone marrow recipients (Figure 1A), verifying effective bone marrow chimerism. In accordance with a negligible role for the bone marrow compartment in the regulation of total body cholesterol homeostasis, plasma total cholesterol levels were not significantly different between Ts65Dn bone marrow and wild-type bone marrow recipient mice, both under chow diet (375 ± 19 and 367 ± 16 mg/dL) and Western-type diet (971 ± 237 and 905 ± 147 mg/dL) feeding conditions (*p* > 0.05). Through combining routine hematological analysis and flow cytometry we measured in our Ts65Dn bone marrow recipients the trisomy-associated lymphocytopenia previously also detected in human Down syndrome carriers (Figure 1B). CD8^+^ cytotoxic T cell concentrations were three-fold lower (*p* < 0.001) in blood of Ts65Dn bone marrow recipients, whilst CD4^+^ helper T cell numbers also tended to be decreased (−32%; *p* = 0.07). The general reduction in blood T cell counts in Ts65Dn bone marrow recipients can probably be attributed to the previously described reduction in common lymphoid progenitors in bone marrow from the trisomic donor mice [14]. Given that, apart from their absolute numbers, the activation state of T cells, i.e., the relative presence of inhibitory natural killer cell receptors [15] and their ability to stimulate secretion of vascular endothelial growth factor [16], is also important in the body’s disease susceptibility, it is interesting to study, in more detail, the T cell phenotypes in Ts65Dn mice and Down syndrome carriers. Bone marrow trisomy was also associated with a marked eight-fold decrease (*p* < 0.001) in blood CD19^+^ B cell concentrations. In accordance with the suggestion of Lorenzo et al. that the B cell deficiency in Down syndrome is due to a diminished proliferation within the spleen [17], the total number of splenic B cells was also >2× lower in Ts65Dn bone marrow recipients (*p* < 0.001; Figure 1C).

**Figure 2:**
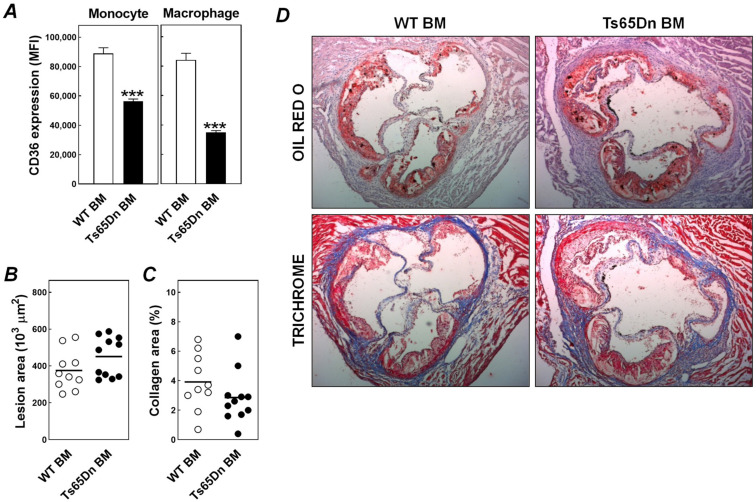
Bone marrow trisomy is not associated with an altered atherosclerosis susceptibility despite a reduced monocyte/macrophage CD36 expression. (**A**) Relative protein expression levels of CD36 in blood monocytes and splenic macrophages, (**B**) aortic root atherosclerotic lesion sizes and (**C**) lesional collagen contents in Western-type diet-fed LDL receptor knockout mice that have received wild-type bone marrow (WT BM) or Ts65Dn bone marrow (Ts65Dn BM). Data in panel *A* represent means + SEM of 6 mice per group. Horizontal lines in panels **B** and **C** indicate respective group averages of the depicted individual mice. *** *p* < 0.001 versus WT BM. Panel **D** shows representative images of atherosclerotic lesions stained for neutral lipids (Oil red O) or collagen (Trichrome; blue staining).

Previous studies have shown that (leukemic) immune cells of human subjects with trisomy 21 are more likely to express CD36 [18]. CD36 is an active contributor to atherosclerotic lesion development as judged from the observation that ablation of CD36 function in macrophages decreases atherosclerosis susceptibility in both hypercholesterolemic LDL receptor and apolipoprotein E knockout mice [19,20]. We therefore performed additional flow cytometric analysis on trisomic and euploid leukocytes, isolated from blood and spleen, to measure relative expression levels of CD36. Interestingly, cell surface CD36 protein expression levels were respectively 37% and 59% lower (*p* < 0.001) in trisomic CD11b^+^Gr1^−^ blood monocytes and F4/80^+^ splenic macrophages as compared to those from euploid bone marrow-transplanted controls (Figure 2A). However, probably as a result of only partial elimination of the macrophage CD36 functionality, this effect did not translate into a bone marrow genotype-associated difference in atherosclerosis susceptibility. Atherosclerotic lesions in the aortic root of Ts65Dn and wild-type bone marrow recipients did not differ in size or their collagen content (Figure 2B–D). It can therefore be suggested that the relative protection against atherosclerosis seen with Down syndrome is rather due to other trisomy-associated changes than those in immune cells derived from the bone marrow. In this context it is interesting to note that Pogribna et al. have shown that plasma levels of the pro-atherogenic molecule homocysteine are reduced in Down syndrome carriers [21]. Total plasma homocysteine levels are similarly reduced in trisomic Ts65Dn mice [22,23]. Notably, the Down syndrome-related hypohomocysteinemia is highly likely not driven by changes to the immune system, as this phenotype can be recapitulated by hepatocyte-specific trisomic overexpression of DYRK1A [24,25].

**Figure 3:**
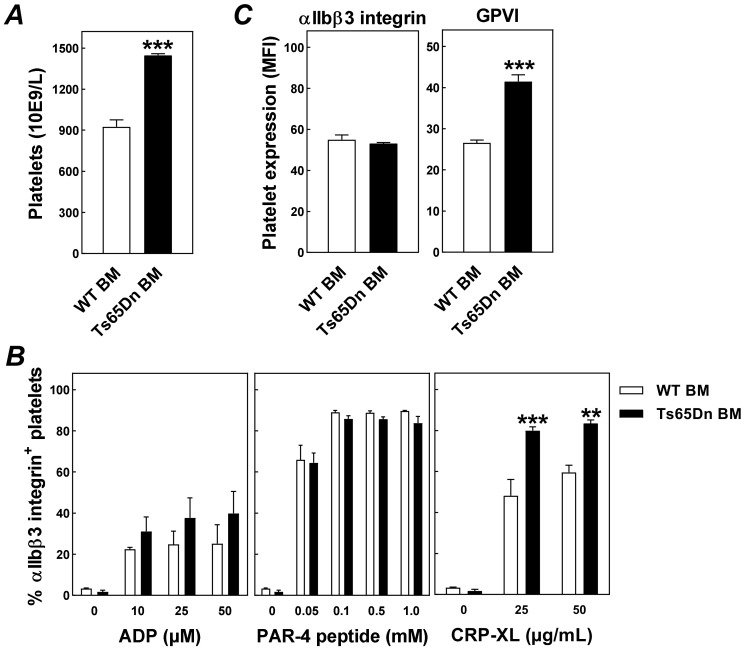
Bone marrow trisomy is associated with thrombocytosis and an exacerbated platelet response to collagen. (**A**) Blood platelet concentrations, (**B**) ex vivo platelet responses to ADP, PAR-4 peptide, and CRP-XL exposure as measured by the presence of active integrin αIIbβ3, and (**C**) platelet protein expression levels of integrin αIIbβ3 and GPVI in Western-type diet-fed LDL receptor knockout mice that have received wild-type bone marrow (WT BM) or Ts65Dn bone marrow (Ts65Dn BM). Data represent means + SEM of 4 mice per group. ** *p* < 0.01, *** *p* < 0.001 versus WT BM.

An interesting finding from our study was that providing mice with a Down syndrome-like bone marrow genotype not only induces lymphocytopenia but is also associated with a significant change in the number of platelets (Figure 3A) and their ability to become activated (Figure 3B). The thrombocytosis observed in Ts65Dn bone marrow recipients was highly likely due to megakaryocyte hyperplasia and extramedullary hematopoiesis, as previously observed by Kirsammer et al. in total body Ts65Dn mice [26]. Bone marrow trisomy did not significantly impact the ADP-induced ex vivo platelet activation, which fits with findings in human platelets from Down syndrome carriers and unaffected controls [27]. The PAR-4-activating peptide-induced platelet response was also normal in Ts65Dn platelets. In contrast, a relatively high fraction of the platelets from Ts65Dn bone marrow recipients expressed active integrin αIIbβ3 upon ex vivo exposure to collagen as compared with platelets from wild-type bone marrow recipients (two way ANOVA: *p* < 0.001 for genotype; Figure 3B). The exacerbated platelet response to collagen can be attributed to an increase in the surface expression of the glycoprotein receptor for collagen GPVI (+35%; *p* < 0.001; Figure 3C), whilst total integrin αIIbβ3 expression levels were not different between the groups of mice (Figure 3C). Notably, since we executed our platelet studies in whole blood specimens, the observed effect on platelet functionality is not necessarily solely resultant from a Ts65Dn platelet genotype, as it may also be secondary to other changes in the blood compartment, i.e., a difference in the concentration of platelet activating factors. However, in light of the finding by Journeycake and Brumley that Down syndrome may be an independent risk factor for developing thromboembolic disease in childhood [28] and other related case reports [29,30], it will be interesting to determine whether our murine platelet findings can be replicated using (washed) platelets obtained from infants and adults with Down syndrome.

## 4. Conclusions

We have shown that transplantation of trisomic bone marrow into LDL receptor knockout mice does recapitulate the lymphocytopenia phenotype of Down syndrome carriers but is not associated with an altered atherosclerosis susceptibility. Our preclinical findings (1) argue against a prominent role for the hematological compartment in the relative atherosclerosis resistance found in human Down syndrome carriers and (2) have uncovered a novel effect of the Ts65Dn-associated trisomy on platelet functionality that can potentially be relevant in human clinical settings.

## Data Availability

All data related to this study that are not already presented in this manuscript are available from the authors upon reasonable request.

## References

[1] Murdoch J.C., Rodger J.C., Rao S.S., Fletcher C.D., Dunnigan M.G. (1977). Down’s syndrome: An atheroma-free model?. Br. Med. J..

[2] Ylä-Herttuala S., Luoma J., Nikkari T., Kivimäki T. (1989). Down’s syndrome and atherosclerosis. Atherosclerosis.

[3] Draheim C.C., Geijer J.R., Dengel D.R. (2010). Comparison of intima-media thickness of the carotid artery and cardiovascular disease risk factors in adults with versus without the Down syndrome. Am. J. Cardiol..

[4] Tansley G., Holmes D.T., Lütjohann D., Head E., Wellington C.L. (2012). Sterol lipid metabolism in down syndrome revisited: Down syndrome is associated with a selective reduction in serum brassicasterol levels. Curr. Gerontol. Geriatr. Res..

[5] Ram G., Chinen J. (2011). Infections and immunodeficiency in Down syndrome. Clin. Exp. Immunol..

[6] Satgé D., Seidel M.G. (2018). The Pattern of Malignancies in Down syndrome and Its Potential Context With the Immune System. Front. Immunol..

[7] Ridker P.M., Everett B.M., Thuren T., MacFadyen J.G., Chang W.H., Ballantyne C., Fonseca F., Nicolau J., Koenig W., Anker S.D. (2017). Antiinflammatory Therapy with Canakinumab for Atherosclerotic Disease. N. Engl. J. Med..

[8] Davisson M.T., Schmidt C., Reeves R.H., Irving N.G., Akeson E.C., Harris B.S., Bronson R.T. (1993). Segmental trisomy as a mouse model for Down syndrome. Prog. Clin. Biol. Res..

[9] Reeves R.H., Irving N.G., Moran T.H., Wohn A., Kitt C., Sisodia S.S., Schmidt C., Bronson R.T., Davisson M.T. (1995). A mouse model for Down syndrome exhibits learning and behaviour deficits. Nat. Genet..

[10] Holtzman D.M., Santucci D., Kilbridge J., Chua-Couzens J., Fontana D.J., Daniels S.E., Johnson R.M., Chen K., Sun Y., Carlson E. (1996). Developmental abnormalities and age-related neurodegeneration in a mouse model of Down syndrome. Proc. Natl. Acad. Sci. USA.

[11] Nahon J.E., Hoekstra M., van Hulst S., Manta C., Goerdt S., Geerling J.J., Géraud C., Van Eck M. (2019). Hematopoietic Stabilin-1 deficiency does not influence atherosclerosis susceptibility in LDL receptor knockout mice. Atherosclerosis.

[12] Hoekstra M., Kruijt J.K., Van Eck M., Van Berkel T.J. (2003). Specific gene expression of ATP-binding cassette transporters and nuclear hormone receptors in rat liver parenchymal, endothelial, and Kupffer cells. J. Biol. Chem..

[13] Aparicio-Vergara M., Shiri-Sverdlov R., de Haan G., Hofker M.H. (2010). Bone marrow transplantation in mice as a tool for studying the role of hematopoietic cells in metabolic and cardiovascular diseases. Atherosclerosis.

[14] Lorenzo L.P., Chen H., Shatynski K.E., Clark S., Yuan R., Harrison D.E., Yarowsky P.J., Williams M.S. (2011). Defective hematopoietic stem cell and lymphoid progenitor development in the Ts65Dn mouse model of Down syndrome: Potential role of oxidative stress. Antioxid Redox Signal..

[15] Costa P., Rusconi S., Mavilio D., Fogli M., Murdaca G., Pende D., Mingari M.C., Galli M., Moretta L., De Maria A. (2001). Differential disappearance of inhibitory natural killer cell receptors during HAART and possible impairment of HIV-1-specific CD8 cytotoxic T lymphocytes. AIDS.

[16] Ciprandi G., Murdaca G., Colombo B.M., De Amici M., Marseglia G.L. (2008). Serum vascular endothelial growth factor in allergic rhinitis and systemic lupus erythematosus. Hum. Immunol..

[17] Lorenzo L.P., Shatynski K.E., Clark S., Yarowsky P.J., Williams M.S. (2013). Defective thymic progenitor development and mature T-cell responses in a mouse model for Down syndrome. Immunology.

[18] Wang L., Peters J.M., Fuda F., Li L., Karandikar N.J., Koduru P., Wang H.Y., Chen W. (2015). Acute megakaryoblastic leukemia associated with trisomy 21 demonstrates a distinct immunophenotype. Cytometry B Clin. Cytom..

[19] Mäkinen P.I., Lappalainen J.P., Heinonen S.E., Leppänen P., Lähteenvuo M.T., Aarnio J.V., Heikkilä J., Turunen M.P., Ylä-Herttuala S. (2010). Silencing of either SR-A or CD36 reduces atherosclerosis in hyperlipidaemic mice and reveals reciprocal upregulation of these receptors. Cardiovasc. Res..

[20] Febbraio M., Guy E., Silverstein R.L. (2004). Stem cell transplantation reveals that absence of macrophage CD36 is protective against atherosclerosis. Arterioscler. Thromb. Vasc. Biol..

[21] Pogribna M., Melnyk S., Pogribny I., Chango A., Yi P., James S.J. (2001). Homocysteine metabolism in children with Down syndrome: In vitro modulation. Am. J. Hum. Genet..

[22] De la Torre R., De Sola S., Pons M., Duchon A., de Lagran M.M., Farré M., Fitó M., Benejam B., Langohr K., Rodriguez J. (2014). Epigallocatechin-3-gallate, a DYRK1A inhibitor, rescues cognitive deficits in Down syndrome mouse models and in humans. Mol. Nutr. Food Res..

[23] Helm S., Blayney M., Whited T., Noroozi M., Lin S., Kern S., Green D., Salehi A. (2017). Deleterious Effects of Chronic Folate Deficiency in the Ts65Dn Mouse Model of Down syndrome. Front. Cell Neurosci..

[24] Noll C., Planque C., Ripoll C., Guedj F., Diez A., Ducros V., Belin N., Duchon A., Paul J.L., Badel A. (2009). DYRK1A, a novel determinant of the methionine-homocysteine cycle in different mouse models overexpressing this Down-syndrome-associated kinase. PLoS ONE.

[25] Latour A., Salameh S., Carbonne C., Daubigney F., Paul J.L., Kergoat M., Autier V., Delabar J.M., De Geest B., Janel N. (2015). Corrective effects of hepatotoxicity by hepatic Dyrk1a gene delivery in mice with intermediate hyperhomocysteinemia. Mol. Genet. Metab. Rep..

[26] Kirsammer G., Jilani S., Liu H., Davis E., Gurbuxani S., Le Beau M.M., Crispino J.D. (2008). Highly penetrant myeloproliferative disease in the Ts65Dn mouse model of Down syndrome. Blood.

[27] Sheppard J.R., Schumacher W., White J.G., Jakobs K.H., Schultz G. (1983). The alpha adrenergic response of Down’s syndrome platelets. J. Pharmacol. Exp. Ther..

[28] Journeycake J.M., Brumley L.E. (2006). Down syndrome as an Independent Risk Factor for Thrombosis in Children. Blood.

[29] Tarlaci S., Sagduyu A. (2001). Cerebral venous thrombosis in Down’s syndrome. Clin. Neurol. Neurosurg..

[30] Kurokami T., Takasawa R., Takeda S., Kurobe M., Takasawa K., Nishioka M., Shimohira M. (2018). Venous thromboembolism in two adolescents with Down syndrome. Turk. J. Pediatr..

